# Reevaluating the role of skeletal muscle in amyotrophic lateral sclerosis pathogenesis: Insights from muscle-derived factors

**DOI:** 10.4103/NRR.NRR-D-25-00567

**Published:** 2025-09-29

**Authors:** Pablo Martinez, Brigitte van Zundert, Fernando J. Bustos

**Affiliations:** Department of Neurology, Baylor College of Medicine, Houston, TX, USA; Institute of Biomedical Sciences (ICB), Faculty of Medicine & Faculty of Life Sciences, Universidad Andres Bello, Santiago, Chile; Millennium Nucleus of Neuroepigenetics and Plasticity (EpiNeuro), Santiago, Chile

Amyotrophic lateral sclerosis (ALS) is a progressive neurodegenerative disease marked by motor neuron (MN) degeneration, neuromuscular junction disruption, and muscle atrophy, ultimately leading to paralysis and death. Despite extensive research, no effective treatment exists, highlighting the need to elucidate mechanisms driving ALS pathogenesis.

About 90% of ALS cases are sporadic ALS and lack a clear genetic cause; the remaining 10% are familial ALS, associated with mutations in over 25 genes. The most common mutations are in superoxide dismutase 1 (*SOD1*) and C*9ORF72*, with rarer variants in *FUS*, *TARDBP*, *TBK1*, and *VCP*. Studies often focus on familial ALS due to its defined genetic drivers, and various *SOD1* mutations have been characterized using rodent models that replicate key disease features (Picher-Martel et al., 2016; Suzuki et al., 2023). While ALS is predominantly considered a neuron-centric degenerative disorder, accumulating evidence supports the contribution of non-neuronal cells, including astrocytes, microglia, oligodendrocytes, and peripheral tissues such as muscle, to disease progression through non-cell-autonomous mechanisms (Harten et al., 2021; Martínez et al., 2024). For instance, previous studies involving the selective expression or knockdown of mutant *SOD1* in specific cell types indicate that astrocytes contribute to key aspects of MN degeneration, locomotor deficits, and reduced survival (Yamanaka et al., 2008). Additionally, astrocyte-driven MN degeneration is associated with several pathogenic events, including oxidative stress, mitochondrial dysfunction, and activation of cell death signaling pathways. In this astrocytic context, we previously demonstrated that excessive inorganic polyphosphate (polyP) released by ALS astrocytes triggers MN death and increases neuronal excitability and Ca^2+^ transients. PolyP-mediated MN degeneration is also accompanied by several other pathogenic events, including oxidative stress and induction of cell death signaling (Arredondo et al., 2022).

In addition to astrocytes, studies in patients and animal models indicate that cells outside the central nervous system are also affected in ALS, including skeletal muscle (Dobrowolny et al., 2009). Specifically, emerging perspectives have shifted the research focus toward understanding the role of muscle tissue in the progression of ALS. Muscle fibers, traditionally considered passive targets of MN activity, are now being recognized as active participants in the disease process, even though their involvement in ALS pathophysiology remains controversial. Early studies suggested that muscle played a minor or consequent role in ALS pathogenesis. For example, MN survival remained unaffected when cells were treated with media conditioned by myocytes expressing mutations in human SOD1 (mutSOD1) (Nagai et al., 2007). Another study using RNAi to reduce mutSOD1 expression in muscle reported no effect on disease onset or survival; however, in contrast to these findings, muscle-restricted expression of mutSOD1 was sufficient to induce ALS-related pathological changes and recapitulate a classic ALS mouse phenotype (Dobrowolny et al., 2009), resulting in severe muscle atrophy, neuromuscular junction abnormalities, and MN axon degeneration, underscoring a potential muscle-derived contribution to ALS pathology (Dobrowolny et al., 2009; Wong and Martin, 2010). While genetic manipulation of muscle in ALS models has provided critical insights into its contribution to non-cell autonomous mechanisms, recent evidence suggests that the role of muscle extends beyond structural dysfunction. Emerging studies indicate that muscle actively secretes molecules capable of exacerbating MN degeneration (Maimon et al., 2018; Martínez et al., 2024). This novel mechanism raises the possibility that muscle contributes to neurotoxicity, potentially affecting motor neuron survival via secreted factors such as inflammatory cytokines, misfolded proteins, and molecules that induce oxidative stress.

**Figure 1:**
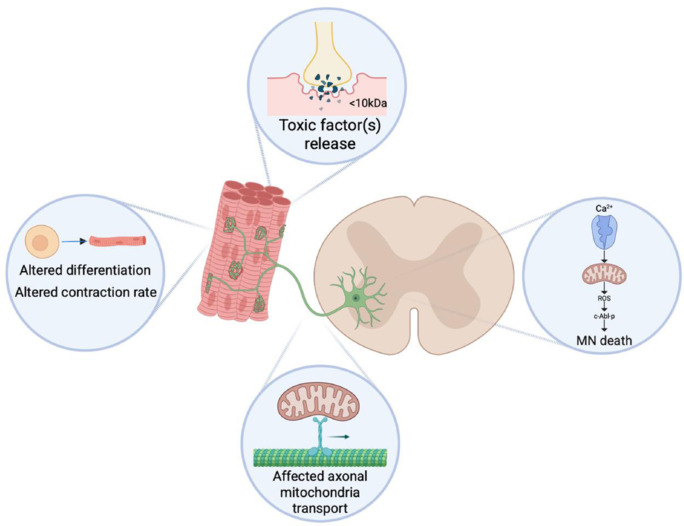
Toxic factors from ALS muscle cells contribute to motor neuron degeneration via multiple pathogenic mechanisms. In ALS, skeletal muscle contributes to MN pathology through a significant non-cell-autonomous pathway that challenges the traditional central nervous system-centric view. The process originates with cell-autonomous defects within the muscle itself, where myotubes, particularly those expressing ALS-linked mutations such as SOD1, exhibit intrinsic dysfunctions such as altered expression of myogenic markers leading to abnormal differentiation and an increased spontaneous contraction frequency. These dysfunctional muscle cells then release soluble, neurotoxic factors into the extracellular space. These factors are characterized as being small molecules, less than 10 kDa in size, and are likely composed of metabolites, short peptides, or amino acids. Once released, these toxic signals exert a multi-faceted assault on the motor neuron. One major effect is the impairment of critical axonal functions, specifically the disruption of mitochondrial transport along microtubules, which starves the neuron of energy and contributes to the decay of the neuromuscular junction. Simultaneously, these factors trigger a lethal cascade within the MN cell body, initiated by the disruption of calcium homeostasis (Ca²⁺ influx). This leads to mitochondrial overload and the subsequent excessive production of damaging ROS. The resulting oxidative stress activates downstream pro-apoptotic signaling pathways, including the phosphorylation and activation of the tyrosine kinase c-Abl, which ultimately culminates in the execution of cell death programs and the degeneration of the motor neuron. ALS: Amyotrophic lateral sclerosis; c-Abl-p: phosphorylated c-Abl; MN: motor neuron; ROS: reactive oxygen species; SOD1: superoxide dismutase 1.

Our recent study also challenges the central nervous system-centric view by demonstrating that skeletal muscle also plays a crucial role in MN degeneration (Martínez et al., 2024; **Figure** **1**). We generated highly homogenous cultures of primary myotubes from mutSOD1 (hSOD1^G93A^) and control WT mice to investigate ALS-associated muscle phenotypes in isolation from other cell types such as MNs, astrocytes, and microglia. We found that mutSOD1 myotubes display intrinsic defects, including altered expression of myogenic markers and impaired contractile function. Our *in vitro* findings — free from MN influence — support a cell-autonomous action of mutSOD1 in muscle cells, consistent with earlier work showing disrupted myogenesis in ALS models (Stella et al., 2023). Functionally, we report increased spontaneous contraction frequency in mutSOD1 myotubes, a finding distinct from previous reports that focused on electrically evoked contractions in adult muscle cells or MN co-cultures. These findings suggest that early, intrinsic alterations in myotube excitability may represent an early event preceding overt neuromuscular disconnection.

Strikingly, conditioned media from mutSOD1 myotubes (mutSOD1-MCM) induced robust MN toxicity of spinal cord cultures. This confirms that soluble toxic factors released by ALS muscle can induce MN death (Camerino et al., 2019; Martínez et al., 2024). The work by Camerino et al. (2019), for instance, demonstrated that muscle-specific expression of mutant SOD1 in an animal model causes sarcolemma hyperexcitability by directly impairing CIC-1 chloride channel function. This intrinsic muscle channelopathy, combined with the reduced expression of the protective, muscle-secreted peptide irisin, establishes a mechanism by which the muscle itself can increase motor neuron vulnerability. Interestingly, the toxicity appears to be mediated by small (< 10 kDa) molecules, likely including short peptides, metabolites, or amino acids — findings that align with recent metabolomics studies of ALS muscle cells (Stella et al., 2023; **Figure 1**). Although polyP has been implicated in astrocyte-mediated toxicity in ALS, we have not yet detected polyP in mutSOD1-MCM, and its presence in this context remains uncertain. Nevertheless, future work should aim to identify these factors and determine their molecular targets. Application of mutSOD1-MCM to MN cultures triggered hallmark ALS pathogenic mechanisms, including axonal mitochondrial transport defects, oxidative stress, and disturbed calcium homeostasis. These events mirror those seen in MNs exposed to astrocyte-conditioned media (Arredondo et al., 2022), suggesting convergent neurotoxic pathways arising from different non-neuronal sources. While our findings support a cell-autonomous role for mutSOD1 in skeletal muscle and demonstrate the neurotoxicity of muscle-derived soluble factors, the study is limited by its *in vitro* nature. Primary myotube cultures, though highly controlled, do not fully recapitulate the whole pathology. Additionally, while we identify toxicity associated with small molecules in mutSOD1-MCM, the precise identity and *in vivo* relevance of these factors remain unknown. Further studies employing *in vivo* models and proteomic/metabolomic profiling will be essential to validate these findings and uncover the mechanistic basis of muscle-to-MN signaling in ALS.

A recent set of data favoring the contribution of non-cell autonomous toxicity of muscle-secreted factors on ALS demonstrated that myocytes express and release Sema3A, which leads to delayed axon growth, promotes axonal degeneration, and disrupts neuromuscular junctions, particularly when paired with MNs carrying ALS mutations (Maimon et al., 2018). However, its role is complex; while Sema3A induces axonal degeneration when secreted from muscle cells, it may act as a trophic factor when expressed by spinal astrocytes. Another recent study showed an altered secreted metabolome of mutSOD1 myocytes (Stella et al., 2023). Interestingly, the authors showed that the secretome of healthy WT myocytes restores defective myogenesis in mutSOD1 primary myocyte cultures, highlighting the role of the muscle secretome in ALS pathogenesis. This finding provides evidence that muscle dysfunction in ALS is, at least in part, reversible and influenced by extracellular signaling rather than intrinsic defects alone.

The role of peripheral tissues in neurodegeneration is not exclusive to ALS. Similar muscle-derived contributions have been proposed in other neurodegenerative disorders, reinforcing the concept of systemic influences in neuronal pathology. For instance, spinal muscular atrophy studies indicate that muscle-secreted factors can influence MN survival and disease progression, drawing parallels to ALS pathology (Fayzullina and Martin, 2014). In Parkinson’s disease, muscle fiber atrophy, altered composition, and even α-synuclein aggregation within muscle have been observed, contributing to motor symptoms and potentially facilitating pathology spread (Duranti and Villa, 2024). Likewise, in Alzheimer’s disease, mitochondrial dysfunction and atrophy in skeletal muscle may exacerbate cognitive decline by promoting systemic metabolic dysregulation. These observations highlight shared pathological features across neurodegenerative diseases, including mitochondrial impairment, protein aggregation, and inflammation, which also manifest in muscle. In Huntington’s disease, altered muscle metabolism and atrophy have been linked to disease progression, further supporting the idea that peripheral tissues contribute to neurodegenerative processes. As recently reviewed (Duranti and Villa, 2024), understanding how muscle tissue actively contributes to disease progression across disorders may open new therapeutic avenues aimed at restoring the metabolic and proteostatic balance systemically. These comparative perspectives underscore the importance of adopting an integrative, non-neurocentric framework when studying ALS and related diseases.

Despite these advances, critical questions remain. How do muscle-secreted factors interact with MNs to promote degeneration? Could modulating these pathways mitigate disease progression? Given the multifactorial nature of ALS, targeting muscle-derived toxic factors such as pro-inflammatory myokines, miRNA, dysregulated proteins, alongside other non-cell-autonomous contributors, may offer a promising therapeutic approach. Targeting muscle toxic factors or enhancing MN resilience could help mitigate disease progression. One approach involves inhibiting toxic muscle-derived factors using neutralizing antibodies or small-molecule inhibitors to block inflammatory cytokines or misfolded proteins secreted by muscle cells. Another strategy focuses on strengthening MN defenses by upregulating antioxidant pathways, enhancing calcium buffering, or boosting mitochondrial function through pharmacological or genetic interventions. Restoring mitochondrial function and axonal transport is another promising avenue, as energy deficits and oxidative stress contribute to MN degeneration. Therapies that promote mitochondrial biogenesis, improve mitochondrial dynamics, or stabilize microtubule tracks could be beneficial. Additionally, modulating calcium homeostasis by targeting calcium channels or calcium-dependent signaling pathways may help protect MNs from excitotoxicity. Given the role of systemic inflammation in ALS, anti-inflammatory strategies could also play a crucial role. Drugs targeting specific inflammatory pathways or immune-modulating agents may help reduce neuroinflammation, ultimately preserving MN function and slowing disease progression.

The accumulating evidence for active role of skeletal muscle in ALS pathogenesis challenges the long-standing central nervous system-centric framework of the disease. Rather than serving as a passive target of motor neuron degeneration, muscle tissue emerges as a dynamic contributor that can influence disease onset, progression, and severity through the secretion of neurotoxic factors and the disruption of neuromuscular homeostasis. These findings underscore the importance of expanding our research to include peripheral tissues in ALS research. As muscle-derived factors contribute to key disease mechanisms, such as mitochondrial dysfunction, oxidative stress, and calcium dysregulation, they represent a promising and not-fully explored therapeutic axis. It will be critical to identify muscle-secreted molecules, elucidate their molecular targets in motor neurons, and determine whether these pathways can be modulated to halt or reverse neurodegeneration. Broadening the scope of ALS research to include the muscle-to-neuron axis not only enriches our understanding of disease biology but may also reveal new, system-level therapeutic opportunities that transcend the limitations of current neurocentric approaches.

*This work was supported by ANID Fondecyt Regular 1250955 (to FJB), ANID Fondecyt Regular 1221745 (to BvZ), UNAB DI-06-24/REG (to FJB), and ANID MILENIO (NCN2023_32, to BvZ and FJB).*
